# Emerging roles of the cellular prion protein (PrP^C^) and 37/67 kDa laminin receptor (RPSA) interaction in cancer biology

**DOI:** 10.1007/s00018-023-04844-2

**Published:** 2023-07-15

**Authors:** Adriana Limone, Valentina Maggisano, Daniela Sarnataro, Stefania Bulotta

**Affiliations:** 1 Department of Molecular Medicine and Medical Biotechnology, University of Naples “Federico II”, Via Pansini 5, 80131 Naples, Italy; 2 Department of Health Sciences, University “Magna Graecia” of Catanzaro, Campus “S. Venuta”, 88100 Catanzaro, Italy

**Keywords:** Autophagy, Cancer hallmarks, DNA damage response, Genomic instability, Protein interaction, Prion-like

## Abstract

The cellular prion protein (PrP^C^) is well-known for its involvement, under its pathogenic protease-resistant form (PrP^Sc^), in a group of neurodegenerative diseases, known as prion diseases. PrP^C^ is expressed in nervous system, as well as in other peripheral organs, and has been found overexpressed in several types of solid tumors. Notwithstanding, studies in recent years have disclosed an emerging role for PrP^C^ in various cancer associated processes. PrP^C^ has high binding affinity for 37/67 kDa laminin receptor (RPSA), a molecule that acts as a key player in tumorigenesis, affecting cell growth, adhesion, migration, invasion and cell death processes. Recently, we have characterized at cellular level, small molecules able to antagonize the direct PrP^C^ binding to RPSA and their intracellular trafficking. These findings are very crucial considering that the main function of RPSA is to modulate key events in the metastasis cascade. Elucidation of the role played by PrP^C^/RPSA interaction in regulating tumor development, progression and response to treatment, represents a very promising challenge to gain pathogenetic information and discover novel specific biomarkers and/or therapeutic targets to be exploited in clinical settings. This review attempts to convey a detailed description of the complexity surrounding these multifaceted proteins from the perspective of cancer hallmarks, but with a specific focus on the role of their interaction in the control of proliferation, migration and invasion, genome instability and mutation, as well as resistance to cell death controlled by autophagic pathway.

## Introduction

**Fig. 1.**
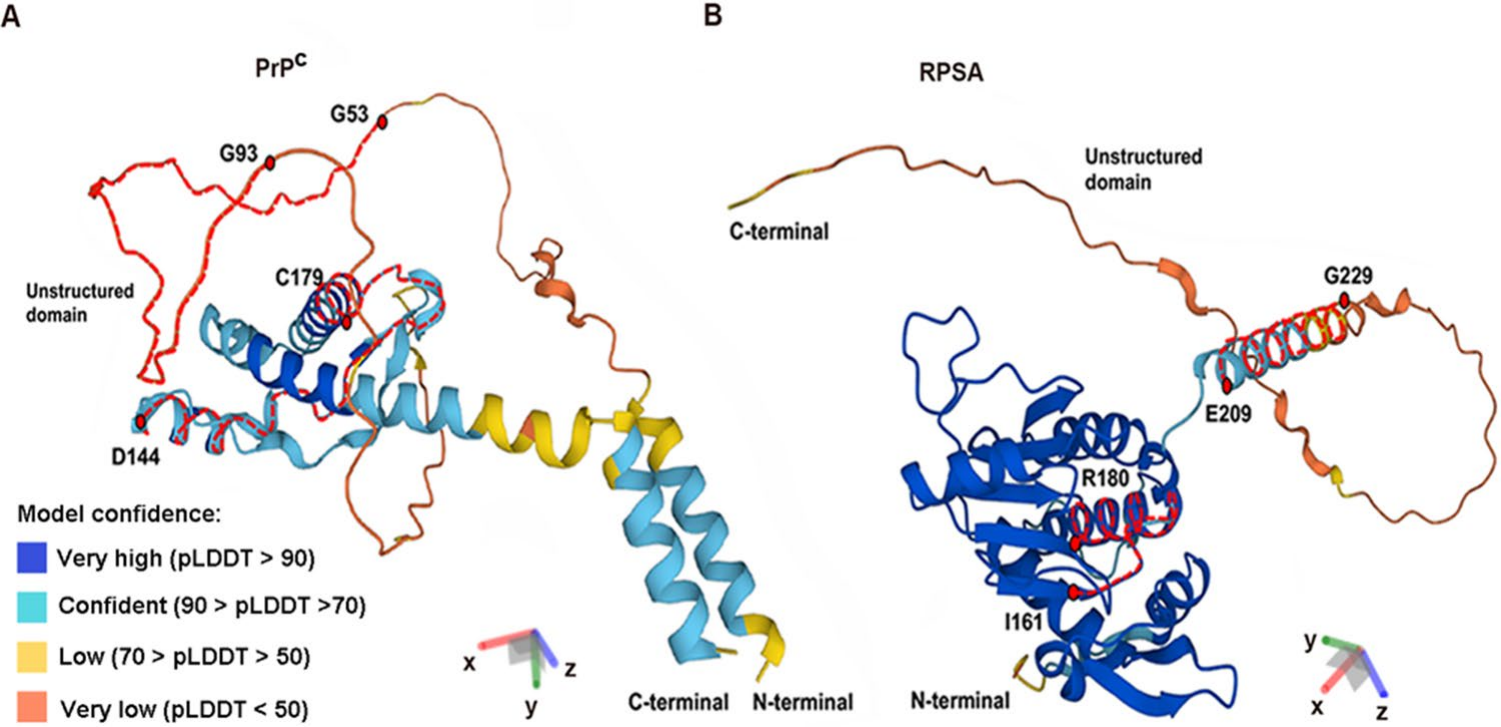
3D models of the full length human PrP^C^ and RPSA proteins. **A** PrP^C^ structure consists of N-terminal unstructured domain and C-terminal structured domain. The dashed red lines indicate two regions on PrP^C^ involved in RPSA binding: the 53–93aa region as an indirect HSPG-dependent interacting domain and the 144–179aa region as direct interaction domain. **B** RPSA structure consists of a globular N-terminal domain and an intrinsically disordered, unstructured C-terminal domain. The two main binding sites for PrP^C^ are indicated in the figure with dashed lines (in red), namely, the 161–180aa and 209–229aa region. To generate 3D models AlphaFold software was used producing a per-residue confidence score (pLDDT) between 0 and 100. Protein regions below 50 pLDDT may be unstructured in isolation

The cellular prion protein (PrP^C^) is a cell surface glycoprotein mainly expressed in central nervous system. Nevertheless, it is ubiquitously expressed in various organs and systems of all known mammals [1]. PrP^C^ has been intensively studied for its involvement in a group of neurodegenerative disorders, known as prion diseases and characterized by misfolding of PrP^C^ into the pathological isoform PrP scrapie (PrP^Sc^) [2]. PrP^C^ is 253 amino acid residues long and it is composed of a “disordered” unstructured N-terminal region and a “structured” C-terminal domain (Fig. 1A). Features of N-terminal domain are an octapeptide repeat involved in the binding of divalent ions, a central hydrophobic tract and two cleavage sites (α and β); the structured domain consists of three α-helices and two short antiparallel β-sheet strands [3, 4].

PrP^C^ undergoes various types of post-translational modifications, among which attachment of a glycosyl–phosphatidylinositol (GPI) moiety, that allows PrP^C^ anchoring to the outer leaflet of the plasma membrane, and several proteolytic processes that generate various truncated or soluble forms of the protein [3, 5]. Altogether, glycosylation and proteolytic processes generate a variety of isoforms that may underlie the wide PrP^C^ functions, ranging from neuronal development to peripheral nerve myelin maintenance, differentiation, stress protection, mitochondrial homeostasis, and cell adhesion. As a GPI-anchored protein, PrP^C^ is largely dependent on its receptors to mediate the aforementioned functions. Among these receptors, a high binding affinity for PrP^C^ is shown by the 37/67 kDa laminin receptor (LR), originally identified as a non-integrin cell surface receptor for laminin-1 (LM), the major component of basement membranes. It was also known as ribosomal protein SA (RPSA), since it was discovered as a component of the 40S ribosome [6]. Afterwards, this protein has been identified as an interactor for both the cellular PrP^C^ isoform and the pathological misfolded PrP^Sc^ [7, 8].

The human RPSA gene encodes for the 37 kDa LR, consisting of a globular N-terminal domain and of an intrinsically disordered C-terminal domain (Fig. 1B). The 37 kDa LR is considered the cytosolic precursor of the higher molecular weight species included the 67 kDa LR; nevertheless, it is still unclear what post-translational modifications are responsible for the conversion of 37 kDa LR precursor into the higher molecular weight isoforms. Interestingly, the 67 kDa LR is a type II transmembrane protein that consists of three domains: the intracellular N-terminal domain, a transmembrane domain, and an extracellular C-terminal domain. The N-terminal domain interacts with the TGF-inhibited membrane-associated protein (TIMAP), thus representing a possible mechanism modulating several downstream signal transduction pathways. The 161–180aa region of RPSA, also known as peptide G, serves as binding site for LM, heparan sulphate proteoglycans (HSPG) and PrP^C^, as well as the 209–229aa region that similarly binds LM and PrP^C^ (Fig. 1B). Mapping analysis in yeast two-hybrid system and cell-binding assay identified two sites on PrP^C^ involved in the interaction with RPSA. Interestingly, the 144–179aa region, also termed PrPLRPbd1, mediates a direct interaction between PrP^C^ and RPSA, whereas the 53–93aa region, PrPLRPbd2, mediates an indirect HSPG-dependent interaction [9].

The structural versatility of RPSA correlates with its multifunctionality, indeed its functions are surprisingly diverse including the well-known cell anchoring via laminins, as well as ribosomal biogenesis and participation to translation, pre-rRNA processing, roles in cellular migration, invasion, viability and growth, cytoskeleton organization, chromatin and histone binding (reviewed in DiGiacomo and Meruelo) [10].

Beyond their physiological roles, recent findings revealed the involvement of both PrP^C^ and RPSA in pathological condition, such as cancer [8, 10–12].

While the relationship between PrP^C^ and the pathogenesis of prion disorders has been largely established, the link between PrP^C^ and cancer progression was first reported when PrP^C^ gene (*PRNP*) was identified as one of the most expressed in pancreatic cancer (PC) cells [13]. Afterwards, PrP^C^ over-expression has been associated with diverse types of solid cancers, such as colorectal cancer (CRC) and glioma [12]. PrP^C^ acts as a key factor to enhance the malignant properties of tumors, being involved in several aspects of cancer biology and sustaining many cancer hallmarks. For instance, PrP^C^ enhances tumorigenesis promoting proliferative signaling, evasion from growth suppressors, resistance to cell death, angiogenesis, migration and metastasis, metabolic reprogramming, evasion from immune surveillance and genome instability. Data relating to the biology of PrP^C^ in tumor, according to each hallmark of cancer, are well-summarized in three recent reviews [14–16]. Increasing evidence demonstrated that PrP^C^ contributes to tumorigenesis via multiple pathways, such as phosphatidyl-inositol-3 kinase (PI3K)-Akt, extracellular signal regulated kinase (ERK) and Notch, by regulating tumor growth, differentiation, and resistance to conventional therapies. In addition, PrP^C^ has been shown to protect glioma cells from autophagic cell death by targeting mammalian target of rapamycin (mTOR) activity [17]. This finding has prompted us to report about the involvement of PrP^C^ in the autophagic pathway related to cancer.

Recently, 48 somatic mutations in *PRNP* were identified in cancer patients [18], among which 8 were reported to be pathogenic for prion diseases, and that cancer patients carrying mutations of *PRNP* may produce PrP^Sc^ and may not be diagnosed with prion disease. From these findings it is likely that both the physiological and the pathological form of PrP^C^ can play a role in the biology of cancer.

Interestingly, many human PrP^C^ variants carrying a pathogenic mutation are trapped in intracellular compartments and have an impaired transport to the cell surface [19–21]. In addition, PrP^C^ mutants associated with Gerstmann–Straussler–Scheinker human prion disease, seem to affect the cytoskeleton tubulin expression, inducing its down-regulation and disruption of microtubules structures [22]. These latter are crucial not only for intracellular protein transport but also for chromosome segregation during cell division. Thus, the variant amyloidogenic forms of PrP^C^, through impairment of cytoskeletal proteins, might influence chromosome disjunction with consequences on genome ploidy and stability, which represent one of the cancer hallmarks.

However, it is still unknown whether PrP^C^ mutants can have an impaired trafficking in cancer cells and whether they are able, as compared to wild-type PrP^C^, to establish interaction with RPSA.

Similar to PrP^C^, RPSA expression has been found increased in neoplastic cells, as compared to their normal counterparts, and directly correlated with several hallmarks of cancer, such as maintenance of cell viability, increase of cellular proliferation, as well as enhanced invasive and metastatic potential [10].

One of the first papers showing that both PrP^C^ and its interactor RPSA were upregulated in cancer, derives from data by Zhou et al. in gastric cancer (GC) [23]. These authors suggested that PrP^C^ and RPSA synergically act to influence GC biology in a manner that patients with high level of both proteins had the poorest prognosis (see the section “Proliferative signaling, invasion and metastasis mediated by PrP^C^/RPSA interaction”).

Moreover, at a cellular level, an interaction between PrP^C^ and RPSA was first established in a two-hybrid screening of a HeLa cDNA expression library for PrP^C^-interacting proteins [9]. The pathogenic significance of PrP^C^/RPSA interaction has been revealed in tissues and cells from scrapie-infected *versus* non-infected mice [24]. In particular, in the brain tissue, high levels of PrP^Sc^ accumulation corresponded to high levels of RPSA, which have been then described to play a crucial role in prion propagation. Although the important role of RPSA in mediating binding and internalization of PrP^C^ and its involvement in pathological mechanisms of prion diseases has been clearly demonstrated [25, 26], the specific role of the cross-talk between PrP^C^ and RPSA in cancer has not been addressed yet, and requires further investigation. Since growing evidence is demonstrating that both these proteins have per se a role in carcinogenesis, our aim in this review is to shed light on the hypothesis that they could synergically modulate the biological processes responsible for the neoplastic transformation, thus representing valid therapeutic targets. Interestingly, Pesapane et al. [27] identified small molecules, targeting the peptide G domain of RPSA, able to inhibit RPSA interaction with LM in normal human kidney cells. Subsequently, we reported that these small inhibitors impaired RPSA interaction with PrP^C^ in neuronal cells, as well as in non-neuronal cells, antagonizing the direct PrP^C^ binding to RPSA via the peptide G domain [28]. These findings are very crucial considering that the main function of RPSA is to enhance tumor cell adhesion to the LM of basement membranes and cell migration, two key events in the metastasis cascade.

The study of the mechanisms underlying the PrP^C^/RPSA interaction might help not only in understanding the pathogenesis of different cancers but also for developing prophylactic and therapeutic measures, as well as for diagnosis.

The primary aim of this review is to focus on the role of PrP^C^/RPSA interaction in the control of cancer hallmarks, such as proliferation, migration and invasion, genome instability and mutation, as well as resistance to cell death modulated by autophagic pathway. This need derives from highlighting the cancer hallmarks mainly shared by the two proteins.

Moreover, to provide a broad overall view on the topic, we also report more recent findings concerning the individual involvement of the two molecules in the aforementioned hallmarks. We believe that this work can represent the basis for future studies on this topic.

## PrP^C^ and RPSA involvement in proliferative signaling, invasion and metastasis

### ***The role of PrP***^***C***^*** in proliferation***

Several works supported a role of PrP^C^ in contexts outside the brain by regulating myelin homeostasis, immune system processes and cancer progression [4]. An over-expression of *PRNP,* unrelated to age, gender and stage, was initially reported in diverse types of solid cancers notably glioblastoma (GBM), pancreatic ductal adenocarcinoma (PDAC) and lung adenocarcinoma (LAC), CRC, GC and breast cancer (BC) [29, 30]. Later, a series of evidence from in vitro and in vivo experimental models suggested the PrP^C^ involvement in tumor biology, affecting fundamental properties of cancer cells first and foremost a sustained cell growth [31, 32]. The inappropriate cancer cell proliferation results from a deregulation of intracellular networks that in physiological situations orchestrate transient cell growth and division during embryonic development and homeostatic maintenance of tissues. In this section, we only focused on the sustaining proliferative signaling pathways involved in cell viability maintenance without including the apoptotic process, a distinct barrier to aberrant cancer cell proliferation, that falls beyond the focus of this review.

Due to PrP^C^ physiological cellular localization (cell surface, Golgi, nuclear and cytosolic forms), it is not surprising that it modulates a wide range of molecules including cell growth mediators and signal intracellular transducers [4, 33, 34], all potential drivers of altered cell proliferation. The role of PrP^C^ over-expression in cancer cell proliferation and the related molecular mechanisms involved, have been established in several experimental models and addressed in recent reviews [12, 16]. One of the most frequently activated signaling pathways in cancer is the PI3K/Akt/mTOR pathway [35]. In human GC cell line, the ectopic expression of PrP^C^ promoted cellular proliferation, at least partially, through activation of PI3K/Akt pathway. As revealed by western blotting analysis, phosphorylated Akt was markedly up-regulated, an effect inhibited by pharmacological blockade of Akt through LY294002 inhibitor [36]. Moreover, the over-expression of PrP^C^ activated cell cycle machinery up-regulating Cyclin D1 at both mRNA and protein levels, thus enhancing G0/G1 to the S phase transition [36] or G2/M *vs* G1/S phase, as reported for glioma cells [37]. Interestingly, octapeptide repeat region in the N-terminal of PrP^C^ plays a pivotal role in promoting the PrP^C^-dependent proliferative effect in GC cells, indeed its deletion almost completely abolished the cellular proliferation [36]. PrP^C^-dependent proliferation has been well-documented also in human BxPC3 and Panc 02.03 PDAC cell lines [38]. Upon PrP^C^ silencing, the two cell lines proliferated slower than the control cells and when inoculated in nude mice a significant decrease of tumor growth was observed.

Wang and collaborators [39] reported that, at molecular level, PrP^C^ silencing downregulated the levels of Notch signaling pathway (commonly activated in tumor) in pancreatic cancer cells, enhancing Notch1 proteasomal degradation and consistently suppressing its downstream transcriptional factor target, Hes1. It was also observed a reduction of Ki-67, a key marker of cell proliferation.

A modulation of Ki-67 has been previously proven both in GBM patients and U87 GBM cells xenografted tumors by Lopes and collaborators [40]. They also found a link between PrP^C^ and heat-shock protein 70 (Hsp70)–Hsp90-organizing protein (HOP, aka stress inducible phosphoprotein 1, STI1 or STIP1), resulting in a modulation of GBM cell proliferation through ERK1/2 and PI3K pathways.

More recently, it has been investigated the subcellular localization of PrP^C^ in PDAC tissues by electron microscopy: cells with higher expression of PrP^C^ (compared with control), revealed a peculiar stoichiometric increase of the PrP^C^ in the nuclear compartment, suggesting a direct role as a transcriptional modulator of several pathways involved in tumorigenesis [41]. Accordingly to pathology results, PrP^C^ expression in PDAC tissues correlated with cancer stage, confirming its role as marker of aggressiveness [42].

In human PC3 prostate cancer cell line, a correlation between PrP^C^ and the amyloid precursor protein (APP) and its proteolytic fragments (Aβ peptides) has been reported [43]. The use of an antibody that recognizes the 92–110 epitope within PrP^C^, that is known to bind Aβ, reduced the tumor growth in PC3 xenografts mice, suggesting a PrP^C^-dependent tumor-promoting effect of Aβ (see also the section “The role of PrP^C^ in invasion and metastasis”). Moreover, we found that a reduction of Aβ in fibroblasts from patients affected by a genetic form of Alzheimer’s disease, was achieved through modulation of RPSA using a specific small molecule inhibitor [44]. In the context of cancer, our finding is promising if we consider the possibility to modulate RPSA and Aβ levels.

In ovarian cancer (OC) tissues and cell lines, it has been reported a decreased PrP^C^ expression both at mRNA and protein level. Next, in vitro experiments showed that *PRNP* overexpression decreased the proliferation capacity of two OC cell lines suggesting, unlike other types of solid tumor, a potential tumor-suppressive role of *PNRP* [45]. This is the only study currently published in total disagreement with the oncogenic role of the PrP^C^ in other cancer types, suggesting how complicated the function and the regulation of PrP^C^ in cancer can be. However, most of the data of this study were based on three public databases, so further in vitro and in vivo experiments will be needed to consolidate these findings.

In tumor microenvironment (TME) of most solid cancers are included cancer stem-like cells (CSCs) which enable tumor growth and progression [31]. A number of evidence indicate that PrP^C^ sustains CSCs in vitro and in vivo proliferation rate of several cancer cells, such as CRC and GBM as described later [46–48].

### *The role of RPSA in proliferation*

In common with PrP^C^, several investigations have clearly demonstrated an increase of RPSA expression in epithelium-derived cancers, as compared to the corresponding normal tissue [8, 49, 50].

SiRNA mediated RPSA knock-down resulted in a significant decrease of viability in different cancer cell lines, such as BC and oesophageal cancer cells [51], pancreatic cancer and neuroblastoma cells [52], melanoma [53] and CRC cells [54]. Regarding proliferation-promoting processes, the main mechanism first explored in cancer cells expressing high levels of RPSA was apoptosis. Since this mechanism is beyond the focus of our review, we only describe the involvement of RPSA in proliferation by maintaining cell viability and controlling survival pathways.

An alternative proposed mechanism of resistance to programmed cell death by which RPSA maintains cell viability is through the high binding with the Midkine, a heparin binding multifunctional protein, overexpressed in a variety of human carcinoma correlating with a poor prognosis. The complex RPSA-Midkine translocated into the nucleus [55], where it could be involved in the maintenance of nuclear stability and in turn in cellular survival [50, 51]. Interestingly, RPSA was also associated with cell adhesion-mediated drug resistance (CAM-DR) in an in vitro and in vivo model of GC drug-resistance cell line. RPSA stimulated Focal Adhesion Kinase (FAK) phosphorylation and the PI3K/Akt and MEK/ERK1/2 survival pathways [56]. Similar results were observed in multidrug resistance CRC cell line SW480 [57].

### ***The role of PrP***^***C***^*** in invasion and metastasis***

In the progression of tumor with aggressive phenotype, the cells acquire the ability to disseminate into the body by different strategies. The cells become invasive and migratory and via bloodstream and/or lymph nodes colonize ectopic tissues generating metastases. All the process is very complex involving both the action of specific cancer cell intracellular pathways and the support from cells in the TME [32]. In this context, cancer cells activate an intrinsic mechanism, namely, epithelial mesenchymal transition (EMT), through which cells able to migrate from the primary tumor, invade surrounding tissues, and reach distant sites [58]. All these processes have been associated with PrP^C^ overexpression in several types of cancer cells [12, 16, 29].

PrP^C^ localization in lipid rafts of the plasma membrane [59] is consistent with its engagement in cell–matrix interactions, in adhesion and migration, cellular processes in which is also involved filamin A (FLNA), an actin binding protein which links cell mechanics and signaling [60].

PrP^C^ is a secretory protein which possesses the N-terminal signal peptide that is cleaved to generate pro-PrP, whereas at the C-terminus contains the signal peptide for GPI–anchor attachment (GPI–PSS). This signal is then cleaved to generate the GPI-anchored form of PrP^C^. In contrast to GPI-anchored PrP isoform, PrP^C^ exists as pro-PrP in most of the human PDAC cell lines (BxPC-3 and Panc 02.03) [38] and in melanoma M2 cell line [61]. Here, the presence of the uncleaved GPI–PSS allows the protein itself to become an integral transmembrane bound protein, able to establish interaction with β1-integrin and FLNA, with a consequent arrangement of actin cytoskeleton. This event provides a migratory phenotype to these cancer cell lines. More recently, to further investigate how PrP^C^ modulates melanoma biology, the same group reported that pro-PrP promotes cancer cell migration and invasion in in vitro and in vivo models, binding two partners known for their involvement in tumor metastasis, namely, Insulin-like growth factor-1 receptor (IGF-1R) and E3 ligase c-Cbl, and promoting autophagy, which is discussed later [62]. Interestingly, pro-PrP contains its GPI–PSS, a crucial sequence of ten aminoacids, that allows the building of trimeric complex pro-PrP/c-Cbl/IGF-1R, and without which the migratory phenotype is mitigated [62]. In several PDAC cell lines (BxPC-3, Capan-1, Panc10.5, Panc-1) pro-PrP isoform interacts with the transmembrane protein Notch, as well as FLNA. Overexpression of pro-PrP increases Notch expression, enhancing cancer cell migration and invasion ability, an effect that can be blocked by a Notch inhibitor [39]. More recently, a molecular and functional link between PrP^C^ and caveolin 1 (CAV1), the protein coat of caveolar plasma membrane identified as marker of EMT [63], has been reported in human PC3 prostate cancer cells, LoVo and MDST8 colon cancer cell lines [43]. Using cell-based assays, the authors found that PrP^C^ up-regulated the expression of CAV1 and, by proximity ligation assays, that the two proteins colocalized in the two aforementioned colon cancer cells, reminiscent of that reported in 1C11 neuronal cells [4]. Further studies will be needed to strengthen these findings and to understand the role of PrP^C^–CAV1 interaction in the context of cancer. In the same study, the authors disclosed that PrP^C^ controlled the expression of APP and Aβ peptides. Using an antibody that blocked PrP^C^–Aβ interaction, it has been established that Aβ–PrP^C^ signaling regulated pathways involved in the mesenchymal subtype of colon cancer cell line, in agreement with a recent finding that identified melanoma-secreted Aβ as a promoter of brain metastasis [64].

PrP^C^ expression increases lamellipodia formation, protrusions of cell membrane organized by actin filaments and considered motor which pulls the cell forward during the process of cell migration and invasion capability of cultured LAC cell lines. These effects were inhibited when cells were treated with the Janus kinase (JNK) inhibitor SP600125, suggesting that PrP^C^ promotes lung cancer cell migration through upregulation of JNK signaling [30].

During invasion process, it is required the activity of matrix metalloproteinase's (MMPs), enzymes that break down extracellular matrix to permit the passage of cancer cells. PrP^C^ overexpression enhances migration and invasion of GC cell lines via activation both of MMP-11 and mitogen activated protein kinase (MAPK) signaling, one of the principal pathways involved in cancer cell migration [65, 66]. Similar results have been reported in BC cells [67].

Among the components of TME, a key role for the progression of the tumor is played by CSCs. When GBM cells were cultured as neurospheres, they expressed both stemness markers, such as CD133 and high levels of PrP^C^; in addition, the two proteins co-localized in plasma membrane of GBM stem-like cells (GSCs) [48]. In PrP^C^-silenced GSCs the authors observed a decrease of CD133 expression and of cell surface adhesion proteins, such as E-cadherin and integrin α6, resulting in a reduced migration on laminin. Collectively, these results provided that PrP^C^ may stabilize cell adhesion molecules on CSCs cell surface. In the field of CRC, Du and collaborators [46] revealed that PrP^C^ expression defines a subpopulation of CD44-positive CSCs (PrP^C+^CD44^+^) with high liver metastatic capability in orthotopic xenograft models, an effect significantly inhibited by administration of PrP^C^ monoclonal antibodies. PrP^C+^/CD44^+^ cells showed an elongated mesenchymal-like morphology characteristic of the EMT phenotype accompanied by an upregulated expression of mesenchymal genes (N-cadherin and Twist), as well as a downregulated gene expression of epithelial markers (E-cadherin). In a recent work, PrP^C^ silencing attenuated the characteristic molecular landscape of a CRC–CSCs subpopulation with mesenchymal phenotype, through the recruitment of the Hippo pathway effectors YAP and TAZ and the TGFβ pathway, suggesting that PrP^C^ deregulation could contribute to a CSCs phenotype in CRC [68]. Moreover, the same group has identified Integrin Linked Kinase (ILK) as close effector of PrP^C^, whose expression controls, mesenchymal phenotype of CRC–CSCs subtype, sustaining adhesion, migration, as well as invasion [69].

Finally, in the context of intercellular communication, the release of exosomes by cancer cells has been associated with several hallmark features of cancer, influencing tumor growth and metastasis [70]. Under hypoxic conditions, CRC cells secreted PrP^C^-expressing exosomes which were able to significantly increase the expression of CSCs markers, such as ALDH1A, Nanog and Oct4, measured by flow cytometric analysis and to sustain tumor progression in an in vitro model [71].

### *The role of RPSA in invasion and metastasis*

RPSA plasma membrane localization allows its binding to one of the principal components of extracellular matrix, LM, a glycoprotein involved in several biological processes, such as adhesion, migration and growth. Several proteolytic enzymes are activated as a consequence of this binding, resulting in the degradation of the basal lamina and in the following migration and invasion of tumor cells [72, 73]. To investigate metastatic potential conferred by RPSA in malignant melanoma cells, RPSA nontoxic specific antibody, IgG1–iS18, has been used [74]. Flow cytometric analysis revealed that melanoma cells displayed high levels of RPSA on their cell surface and the treatment with IgG1–iS18 antibody determined a significant reduction of the adhesive potential to LM and the invasive capacity of malignant melanoma cells, confirming previous results reported in several types of cancer cells [75–78]. For instance, in A549 lung cancer cell line, downregulation of RPSA with specific siRNA, inhibited cellular migration activity, as evaluated by scratch motility assay [79]. From a mechanistic point of view, proteomic analysis performed in pancreatic cancer cells, revealed that RPSA interacted with Integrin alpha 6 (ITGA6) and that they colocalized on the plasma membrane as showed by immunofluorescence analysis. Interestingly, although RPSA interacted with ITGA6, they promoted cellular migration by activating two different signaling pathways, namely, PI3K and MAPK, respectively [80]. More recently, Gresseu and collaborators [81] reported that neurospheres derived by U87 glioblastoma cell culture, expressed increased levels of RPSA, of EMT genes, such as SNAIL and fibronectin, as well as stemness markers SOX2 and CD133, compared to corresponding 2D culture cells. These data suggested a correlation between RPSA expression both with EMT process and the acquisition of CSC phenotype.

### ***Proliferative signaling, invasion and metastasis mediated by PrP***^***C***^***/RPSA interaction***

The observation that PrP^C^ and RPSA are overexpressed in GC and that the two proteins interact with high affinity [82], led Zhou's group to analyze their expression levels in a cohort of 238 patients affected by GC by combining the tissue microarray technique and immunohistochemical method. It has been also evaluated their correlation with the clinicopathological characteristics of patients to understand if their co-expression could be used as a marker of poor prognosis [23]. They found that tissues with high expression of PrP^C^ also had a high rate of upregulated expression of RPSA and for these patients the prognosis was the poorest. Later, the interaction between PrP^C^ and RPSA has been assessed both by co-localization, examined by immunofluorescence staining, and co-immunoprecipitation experiments in GC cell lines and tissues [83].

The biological effects of PrP^C^/RPSA interaction were for the first time evaluated in the development of Merlin-deficient tumors by Provenzano and collaborators [84]. To demonstrate that the observed PrP^C^ overexpression was due to Merlin deficiency, they reintroduced wild-type Merlin gene in cancer cells with consequent strongly reduction of PrP^C^ levels. The increased PrP^C^ expression was caused by its upregulated transcription and not by decreased proteasomal degradation. PrP^C^ knockdown induced a reduction of Ki67-positive proliferating cells, as well as of cyclin D1, accompanied by a decreased expression and activation of ERK1/2, FAK and Akt. In addition, cancer cells were less capable of adhering to the cell matrix. All these cellular events were mediated by PrP^C^ acting via RPSA [84]. In addition, the same authors demonstrated that PrP synthetic peptide (aminoacid residues 105–120 of the human PrP) was able to protect schwannoma cells from H_2_O_2_-mediated cell stress. This study represents one of the first evidence for signaling of PrP^C^ via RPSA with the engagement of downstream Erk1/2, PI3K/Akt and FAK signaling pathways to control survival, proliferation and cell–matrix–adhesion in schwannoma cells, suggesting PrP^C^ and its interactor as potential therapeutic targets for schwannomas. Moreover, the same group [84] described a strong release of PrP^C^ through exosomes from schwannoma cells and, together with an independent paper from Guitart et al. [85], reporting the presence of both PrP^C^ and RPSA in exosomes deriving from neuronal cells, reinforced the idea to possibly modulate the two proteins for fight cancer.

## Genome instability and mutation: the role of PrP^C^ and RPSA

The aberrant proliferation of cancer cells correlates with both an increased rate of genomic changes and accumulation of mutations in multiple genes regulating cell division and tumor suppression, further conferring advantage to the neoplastic transformation of cells. This cancer hallmark is known as genomic instability [31].

### ***PrP***^***C***^*** in DNA damage response (DDR)***

PrP^C^ has been largely demonstrated to be involved in the maintenance of genomic stability after exposure to oxidative stress and genotoxic stimuli as a key factor in the DDR. The requirement of PrP^C^ to preserve genome stability in response to genotoxic insult, has been shown in mouse brain and in human neuroblastoma cells by Bravard et al. [86]. The results obtained in both experimental models, indicated PrP^C^ as a strong activator of DNA damage repair through stimulation of the base excision repair enzyme apurinic/apyrimidinic endonuclease (APE1) activity in the nucleus. Moreover, genotoxic stress, as well as oxidative stress, leads to transcriptional activation of *PRNP,* further reinforcing the scenario of PrP^C^ involvement in DDR [86–88]. PrP^C^ upregulation has been also reported in response to copper-induced oxidative stress by ataxia telangiectasia-mutated (ATM)-mediated pathway [88]. PrP^C^ was reported as marker of cellular stress to aneuploidy in colon cancer screening by Domingues et al. [89]. In this study, conducted in HCT116 CRC cells upon serum deprivation, the levels of PrP^C^ increased in aneuploid cells and were protective against serum-deprivation induction of death. The main message deriving from this research was that the upregulation of PrP^C^ in aneuploid cells was a consequence of the oxidative stress associated with this genomic alteration. These findings fit well with the data from Bernardino et al. [90], where PrP^C^ expression conferred protective role against irradiation. Collectively, these studies sustain that upon DNA injury, PrP^C^ is induced and its main job is to protect cells from DNA damage, hence it is clearly emerging a role for PrP^C^ in balancing DNA damage and repair [89, 90]. Thus, PrP^C^ has been proposed to represent one of the protein needle of the balance between cell survival and growth, two critical events in cancer cells. In this context, it should be noted that expression of genes for prion protein changes in certain cancers, such as glioblastoma, melanoma, pancreatic and GC [29, 91]. The protective function of PrP^C^ could be crucial to prevent the long-term accumulation of DNA damage in neurons; however, since the ubiquitous expression of PrP^C^ in mammalian tissue, it is likely that DNA protective pathway can be active in other cell types.

### *RPSA in DDR*

Similar to PrP^C^, RPSA receptor is involved in DDR by controlling the nucleolar localization of the double-strand break repair factors RNF8 and BRCA1 [92]. RPSA interacts with the ubiquitin ligase RNF8 in the nuclei of HEK293T cells, whereas upon γ-irradiation, RNF8 dissociates from RPSA and translocates into the nucleoplasm to participates to DNA repair, thus suggesting a putative role for RPSA in RNF8 anchoring to nucleolus. Likewise, BRCA1 nucleolar localization requires both RPSA and RNF8, although their interaction has not been demonstrated yet.

Altogether, these studies highlighted in DDR a physiological role of PrP^C^ and RPSA, which can exert a protective function against the accumulation of DNA damage. In a pathological context, the over-expression of PrP^C^ and RPSA frequently reported in several cancer types, can be the consequence of the genomic instability and it likely represents a mechanism through which the cells enhance the DDR to counteract the accumulation of DNA damage. On the contrary, one could also speculate that the unbalanced expression level of PrP^C^ and RPSA can contribute to genomic instability causing a dysfunctional response to DNA damage and replication stress. Hence, to date, it still remains under debate whether the upregulation of PrP^C^ and RPSA in pathological context could be a mechanism that sustains or counteracts DDR and genomic instability.

### ***PrP***^***C***^*** in chromatin remodeling***

Beyond the recruitment of the repair factors, another important aspect of DDR includes the chromatin remodeling. Although PrP^C^ is a cell surface protein, it is well-established that it can translocate to the nuclei [93], where it shows high affinity for polyanionic molecules, such as nucleic acids (reviewed in Silva et al.) [94, 95]. The ability of PrP^C^ to interact with nucleic acids has been extensively demonstrated both in vitro [96] and in vivo [97], and this interaction can lead to conformational changes of both the protein and the nucleic acid molecule itself. Indeed Bera et al. [96] demonstrated that PrP^C^ is able to bend and unwind DNA, resembling the same structural changes of DNA induced by proteins involved in transcriptional regulation.

Beside the interaction with nucleic acids, it was also demonstrated the interaction of PrP^C^ with chromatin histones, such as H1 and H3 [93], strengthening previous data on PrP^C^ involvement in transcriptional regulation in the nucleus.

### *RPSA in chromatin remodeling*

RPSA as well, potentially interacts with chromatin, both directly interacting with DNA, as demonstrated in in vitro study [98] and more tightly through histones [99]. Hence, among the RPSA binding proteins identified in NIH3T3 cell extracts, there is a large group of proteins involved in DNA/chromatin maintenance. Histones H2A, H3 and H4 were all identified to be interactors of RPSA. In addition, SWI/SNF complex subunit SMARCC1/BAF155, which is thought to regulate transcription through alteration of chromatin structure, was isolated as RPSA interactor [98]. Collectively, these data suggest that the association of RPSA with both histones and chromatin modifying proteins, results in chromatin maintenance or modification.

### ***PrP***^***C***^*** regulating genomic stability***

The aforementioned studies are focused on the involvement of the PrP^C^ isoform in several aspects of carcinogenesis, i.e., DDR and chromatin remodeling. However, to date, we cannot conclude whether amyloid directly or indirectly affect carcinogenesis, since only the expression levels of genes for amyloidogenic proteins and not the presence of amyloids in the cells were considered. PrP^C^, as well as other amyloidogenic proteins, such as amyloid beta peptide (Aβ), tau and α-synuclein, all associated with neurodegenerative diseases, may undergo conformational transitions, during which the content in β-sheet structures increases, and form amyloid oligomers, which are able to grow via interactions between the same regions of protein molecules generating protofibrils [100]. These structures are then combined into fibrils and amyloid aggregates [101]. PrP^C^ in its PrP^Sc^ form, has a β-sheet proportion of about 43%, approximately 14 times greater than in its monomeric form PrP^C^ (3%) [102]. A prion-like prionogenic protein, such as tau and α-synuclein, is able to form different inherited conformational variants of aggregates which possess diverse biological properties known as prion strains [103].

Moreover, as reported in a recent review from Andreychuk et al. [104], studies in yeast and bacteria, suggest that prion and prion-like proteins [100, 105] may play a role in regulating DNA replication, thus being directly involved in maintaining the genome stability [106].

At this point a question arises: how can an amyloid protein-like PrP^C^, affect the stability of the genome in human cells? One possible consideration might be based on the fact that the transition of a protein into the amyloid state seems to affect the microtubules-driven chromosome segregation during mitosis and meiosis [107]. The dynamic instability is a critical point for the function of this class of cytoskeleton filaments. Alteration of microtubules polymerization, as well as spindle-assembly and chromosome segregation, has been associated with the yeast prion protein Sup35p mutations [108]. In mammals, a direct interaction between PrP^C^ and tubulin has been identified in extracts from porcine brains [109], and in vitro experiments using standard light scattering measurements and electron microscopy, showed that full length recombinant PrP^C^ induces a rapid increase in tubulin oligomers associated with sheets of protofilaments, accompanied by reduction of the length of microtubules [110]. These data contributed to postulate that inducing formation of stable tubulin oligomers, PrP^C^ may act as an inhibitor of microtubule assembly. Moreover, PrP^C^ mutants associated with Gerstmann–Straussler–Scheinker human prion disease, seems to yield even a stronger effect on the down-regulation of tubulin expression, with respect to the normal PrP^C^ counterpart, inducing disruption of microtubules structures [22]. It is tempting to speculate that the amyloid forms of the protein, through impairment of cytoskeletal proteins, can cause chromosome nondisjunction during cell division with consequences on genome ploidy.

### *RPSA regulating genomic stability*

A similar interaction with cytoskeletal proteins has been reported for RPSA, as well [111]. RPSA directly interacts with tubulin in NIH3T3 cells acting as a tethering protein, holding the ribosome to tubulin, which is crucial for protein biosynthesis. Moreover, RPSA directly binds to actin, which instead is critical for cell migration. These data indicate that interactions between RPSA and the cytoskeleton, are vital in mediating two cellular processes linked to cancer: protein synthesis and migration. However, whether RPSA interaction with cytoskeleton can influence genome ploidy still remains an open issue.

In this context, another important source of aneuploidy is the cancer-relevant change termed tetraploidization, that has been strictly related to telomere crisis [112]. Telomere-driven tetraploidization occurs in human cells undergoing crisis and promotes transformation of cells [112, 113]. At the ends of the chromosomes, the repetitive DNA sequences known as telomeres, are subjected to the “end replication” problem of DNA polymerases and are maintained by the ribonucleoprotein DNA polymerase complex, telomerase [114]. Telomere crisis occurs during tumorigenesis when depletion of telomere reserve leads to telomere fusion, whose consequence is the formation of dicentric chromosomes, which have been proposed to drive genome instability [115].

In addition to their role in telomeres extension, telomerase and its enzymatic subunit, known as human telomerase reverse transcriptase (hTERT), possess other extra-telomeric functions, such as DNA repair and cell proliferation, which all together converge towards preservation of cell viability [116]. Interestingly, hTERT overexpression and telomerase activity have been detected in most cancer types [117–120], and among the various proteins found to interact and control hTERT/telomerase functioning in human cancer cell lines, the group of Naidoo et al. [121] identified RPSA. The authors postulated that RPSA upregulation may enhance telomerase activity [122, 123]. Reversely, siRNA-mediated RPSA knock-down impeded telomerase activity in breast and lung cancer cells [79, 121]. Likewise, a significant decrease in telomerase activity and hTERT expression levels was observed in late-stage CRC cells via RPSA siRNA technology, resulting in genetic instability and activation of DDR pathway [54, 124].

## Resisting cell death through modulation of autophagic pathway: what about the role of PrP^C^ and RPSA?

Resistance to apoptotic cell death was the first cancer hallmark to be connected to PrP^C^, nearly 20 years ago [125]. The authors described the upregulation of *PRNP* transcripts in adriamycin-resistant GC cells, as compared to the normal counterpart. In various cell types, both apoptotic and autophagic cell death pathways can be induced by serum deprivation. Thus, here we will exclusively report and discuss data on the involvement of PrP^C^ and RPSA in autophagic mechanism, mainly in cancer.

It is important to highlight that in cancer cells, three distinct pathways of active autophagy can be induced by amino acid starvation: chaperone-mediated autophagy (CMA) dependent by mTOR inactivation, selective endosomal microautophagy independent of mTOR inactivation and the canonical, ATG protein-assisted macroautophagy, that can be activated by mTOR-dependent signaling. All the three main autophagy pathways likely work in concert and may partially compensate for each other.

Autophagy, also termed type II programmed cell death, is a lysosome-mediated catabolic pathway that allows the elimination of damaged organelles, misfolded protein, thus contributing to cellular homeostasis in non-transformed cell. In cancer, autophagy has a dual role acting both as tumor suppressor and tumor promoter. In fact, autophagy, by eliminating damaged cells and organelles, counteracts malignant transformation during early tumorigenesis. Conversely, in tumoral cells, the autophagic pathway can sustain the survival of cells in the presence of several stress stimuli (for example, hypoxia) representing a tumor-promoting mechanism. The bipolar role of autophagy in cancer is described in more detail in a recent review [126].

### ***PrP***^***C***^*** in autophagy***

One of the first finding concerning the involvement of PrP^C^ in the autophagic process, has been reported in neuronal cells by Oh et al. [127], which have shown higher autophagic activity in Zürich I mouse *PRNP*^0/0^ hippocampal neuronal cells than in wild-type mouse cells. This mechanism was retarded by the introduction of PrP^C^ into *PRNP*^0/0^ cells, but not by reintroduction of PrP^C^ lacking the N-terminal octapeptide repeat region, suggesting that this protein region plays a critical role in modulating autophagy exhibited by PrP^C^. Moreover, Oh et al. [128] provided the first evidence that the deficiency of PrP^C^ may impair autophagic flux via H_2_O_2_-induced oxidative stress in hippocampal cells.

The evidence about the PrP^C^-mediated modulation of autophagy in neurodegeneration has been well-established; however, many of the mechanisms described in neurodegenerative diseases might be shared with tumorigenesis. Indeed, in recent years, several studies have highlighted the correlation between PrP^C^ and autophagic flux modulation in different tumor types.

In tumor cells, the crucial challenge to depict the crosstalk between PrP^C^ and carcinogenesis, is to identify the proteins interacting with PrP^C^. Among the multiple molecular interactions that PrP^C^ physiologically performs, the ones shared with the autophagic activity are those involved in tumor carcinogenesis of glial cells. Such activity has been identified on mTOR through which PrP^C^ operates for neuroprotection via PI3K/Akt pathways [129].

As reported in the above sections, Akt–PI3K–mTOR signaling axis is among the most frequently deregulated pathway in cancer cells, thus sustaining abnormal proliferation. At the same time, mTOR is considered a master regulator of the autophagic flux through phosphorylation of several substrates.

As described by Akhavan et al. [130], GBM can be considered as a pathology characterized by defects in mTOR activity, at the early stage of its development. mTOR acts both as upstream regulator and as a downstream effector of PI3K. A constitutive PI3K pathway activation is a hallmark of GBM [131].

The role of PrP^C^ in autophagic processes can represent a crucial event in glioma tumorigenesis as reviewed in Armocida et al. [132]. Another report on the ability of PrP^C^ to modulate autophagic cell death in glial tumor cells, comes from studies by Barbieri et al. [17], where PrP^C^ silencing resulted in inhibition of the kinase activity of mTOR, promoting autophagic cell death.

More recently, Lenzi and collaborators [133] provided evidence that rapamycin induced a persistent clearance of PrP^C^ in GBM cells, and removal of PrP^C^ was consistent with enhanced autophagy flux, which efficiently clears such an aggregate prone protein.

As recently reported by Li and collaborators [62], in human PC cell lines and in melanoma M2 cell line, the presence of the uncleaved GPI–PSS in pro-PrP allows the protein to bind the E3 ligase c-Cbl facilitating the IGF-1R ubiquitination for its degradation and consequent autophagic activation through Akt–ULK1 signaling axis, driving melanoma lung metastasis. Importantly, disruption of pro-PrP/c-Cbl/IGF-1R complex using a synthetic peptide, induced a reduction of cancer cell autophagy and mitigated tumor aggressiveness. These findings provide a therapeutic approach for treating human cancers expressing pro-PrP.

### *RPSA in autophagy*

Concerning the role of RPSA in autophagic process, we have recently proposed a pivotal role for RPSA in modulation of autophagic pathway in neuronal cells [134, 135]. The so-called “canonical” autophagic process is orchestrated by the hierarchical and coordinated activity of autophagy-related genes ATG, and the autophagic machinery is modulated by the upstream ULK1/2 complex and the PI3K-class 3/beclin 1 (PI3KC3/BECN1) complex, all together representing the pre-initiation complexes.

Beside this, it is now well-established that ATG proteins may play a role distinct from canonical autophagy and defined as non-canonical pathway driving unconventional microtubule-associated protein 1A/1B-light chain 3 (LC3)-lipidation on endosomes [136–138]. LC3, in its lipidated form, is a protein associated with the membrane of forming autophagosomes, whose source of formation mainly derives from plasma membrane and endocytic vesicles [139].

From previous observations, it is now evident that LC3 lipidation can occur independently of canonical autophagy regulators, involving endosomal compartments [139]. In agreement with the above described role of endosomes in autophagic events, we found that inhibition of RPSA by NSC48478, reflected on the upregulation of a large cluster of endosomal-related genes and was accompanied by the induction of LC3 lipidation on Rab5-positive-endosomes. Our findings indicate that RPSA inhibition stimulates a “non-canonical” autophagic pathway, thus sustaining the hypothesis of RPSA-mediated modulation of autophagic process [134, 135].

Interestingly, RPSA is expressed on the cell surface and is internalized through early endosomes. These compartments resulting from the endocytic pathways, have been shown to generate signaling platforms. However, it is not known whether the early endosomes resulting from the internalization of RPSA can exhibit such cell signaling property.

The YIGSR pentapeptide sequence in the β1 chain of laminin-1 has been revealed as the binding site for RPSA [140], and the administration of LM or YIGSR induced internalization of RPSA into early endosomes of adrenal pheochromocytoma PC12 cells. This caused a sustained generation of cell signaling that increased the survival of cells from death induced by serum starvation [141], which is known to induce autophagic cell death.

Beside the evidence of autophagic modulation through non-canonical route, we and other groups demonstrated the role of RPSA as mediator of cellular pathway including autophagy. Several studies demonstrated the correlation between RPSA and the modulation of Akt–PI3K, as well as MAPK/ERK signaling pathway, which, as described above, are crucial regulators of canonical autophagic pathway. For instance, we demonstrated that the inhibition of RPSA inactivates Akt in human skin fibroblasts from familial Alzheimer’s disease [44].

Moreover, our previous finding concerning the inactivation of MAPK/ERK signaling induced by RPSA inhibition in neuronal cells [142] could be of great interest, if one considers the possibility that non-canonical autophagy could generate endo-lysosomal signaling hubs [143, 144]. As such, this hypothesis might provide insight into the efficacy and function of RPSA inhibitor as an autophagy-modulating drug that possesses the property of endosomal lipidation of LC3.

Similar to the above described effects, A375SM melanoma cells expressing reduced level of LR, showed a significantly increased phosphorylation of ERK, JNK and p38, regardless of the exposure to exogenous laminin [145]. To note, Akt/PI3K and MAPK signaling converges on the regulation of mTOR. Hence, the regulation of growth signals, such as Akt–PI3K and MAPK, links RPSA not only to pro-survival signaling but also to autophagic flux.

To date, the interplay between RPSA and signaling pathways controlling autophagy in the context of tumorigenesis still remains underestimated; however, altogether, the aforementioned studies could help to identify molecular mechanisms that can be in common with a complex pathology, such as cancer.

The effect on autophagy modulation of PrP^C^ linked to its receptor RPSA in cancer is even less investigated. Recently Luo et al. [83] demonstrated that MGr1–Ag/37LRP (that is the precursor of RPSA) may interact with PrP^C^ and promote the PrP^C^-induced multi-drug-resistance in GC through PI3K/Akt pathway. Thus, knockdown of MGr1-Ag/37LRP significantly attenuated PrP^C^ induced multi-drug-resistance by sensitizing drug-induced apoptosis through inhibition of Akt. In this study, the authors explored the contribution of MGr1-Ag/37LRP to PrP^C^ mediated multi-drug-resistance in GC without investigating a possible downstream effect on autophagic pathway. However, in our opinion, this study lays the foundation to further investigation about the hypothesis that PrP^C^ and RPSA could jointly coordinate the autophagic pathway.

Several studies have advanced the hypothesis that diverse signaling pathways controlled by PI3-kinase/Akt, PKC, Fyn and Erk1/2, all implicated in the control of autophagic pathways, are modulated by PrP^C^ expression and interaction with other partners, such as RPSA (reviewed in Mehrpour and Codogno) [33]. In this scenario, such a complex could be destroyed by PrP^C^ toxic mutants (lacking N-terminal region, or the central domain of PrP^C^ [146, 147]) possibly by competing for the binding of some complex components yet failing to interact with the signal transducing factors.

**Fig. 2:**
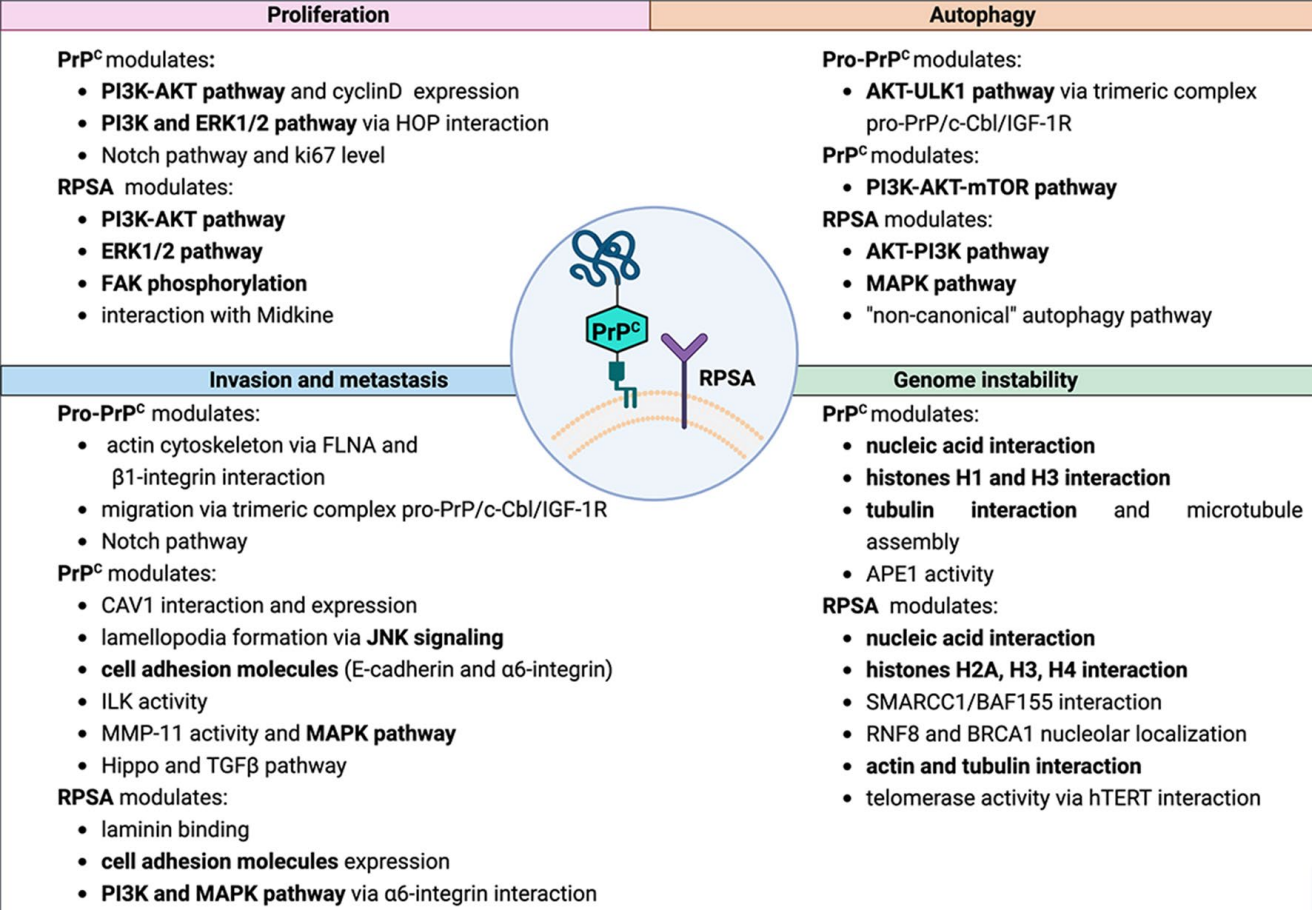
Summary of the data supporting the involvement of PrP^C^ and RPSA in the regulation of several cancer hallmarks. Proteins and signaling pathways modulated by PrP^C^ and RPSA in cancer cells are reported. The features shared by the two proteins related to cancer hallmarks have been highlighted in bold. Scheme created using BioRender.com

A schematic summary of PrP^C^ and RPSA influences on cellular events related to tumorigenesis up to this point discussed, is provided in Fig. 2.

## Concluding remarks

**Fig. 3:**
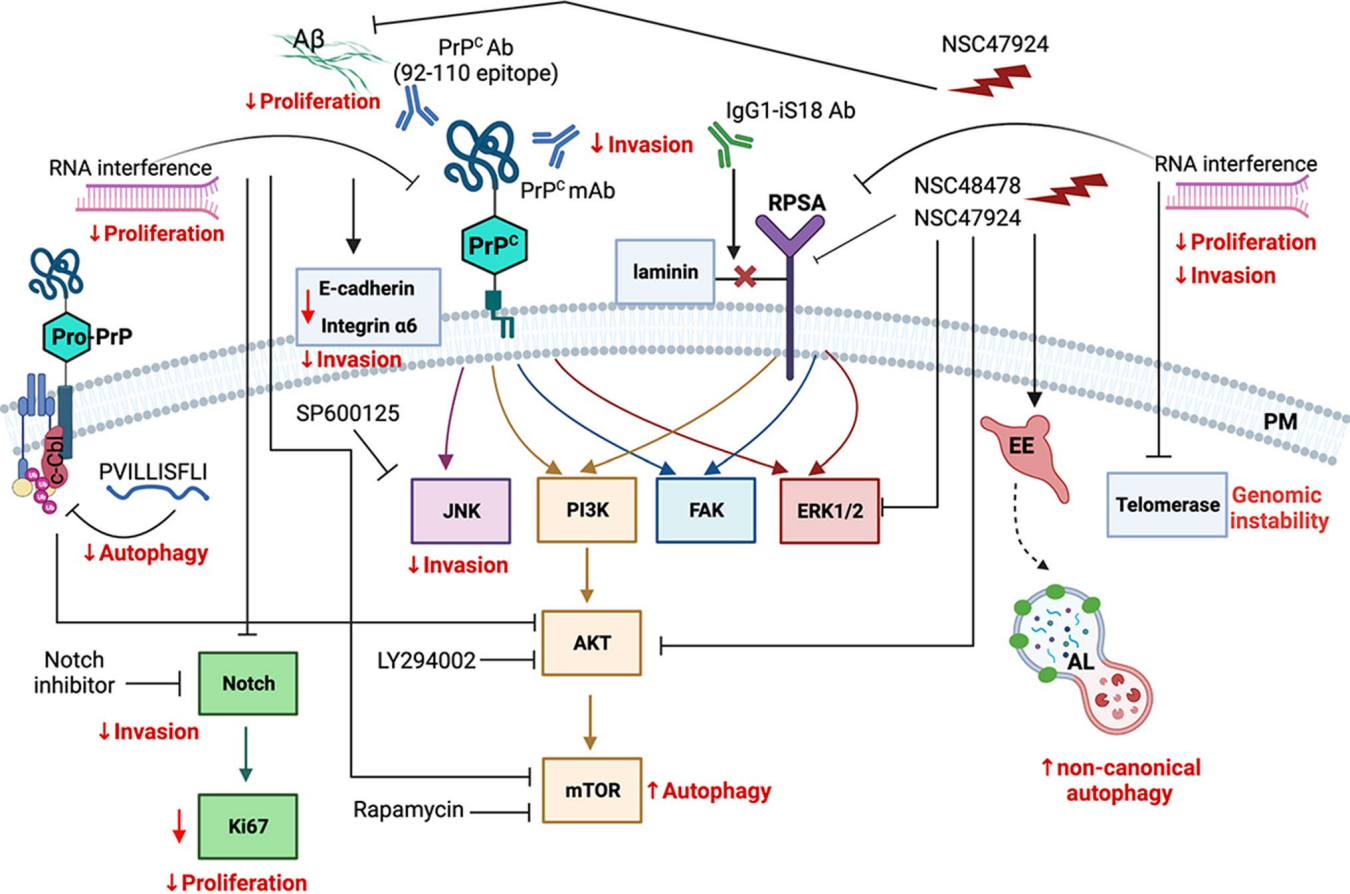
Strategies to modulate tumor progression by regulating PrP^C^ and RPSA expression and function. Cancer hallmarks affected by targeting PrP^C^ and RPSA (RNA interference techniques, antibodies or inhibitors) have been reported in red. Scheme created using BioRender.com

Beyond the undoubted involvement of PrP^C^ in neurological disorders [2], many proofs in recent years showed that it is overexpressed in diverse solid tumors and plays a role in the onset and development of cancer influencing cellular events, such as genome instability with consequent gene mutation, proliferation, migration and invasion together with resistance to autophagic death [16, 148]. Among its wide number of ligands, PrP^C^ interacts with RPSA protein, a versatile non integrin LM receptor whose overexpression in solid and haematological malignancies has been associated with the potential invasive and metastatic cell phenotype [10, 50]. Compelling data suggest that PrP^C^ and RPSA can be considered on their own as potential therapeutic targets [12, 149] and Fig. 3 provides a strong visual support to this idea.

Silencing of PrP^C^ expression through antisense oligonucleotide-based strategies could be helpful in the field of cancer therapy, as reported for certain type of tumors [48, 148]. Recently, pro-PrP has been pinpointed as potential therapeutic target to reduce melanoma metastasis in vitro and in vivo, using a peptide that inhibited GPI–PSS of pro-PrP and some of its interacting partners [62]. Application of monoclonal antibodies against PrP^C^ in preclinical models of cancer cells, such as mesenchymal colon cancer [43] or CSCs–CRC [46], has proven to be a well-founded strategy to mitigate cellular aggressive phenotype with minimal toxicity.

RPSA interaction with LM, the main extracellular matrix glycoprotein, makes the receptor a promoter of invasive and metastatic characteristics of cancer cells. Specific antibodies for RPSA and siRNAs targeting RPSA are two therapeutic strategies employed in many tumorigenic in vitro cell lines and both effective in inhibiting the characteristics of multiple cancer hallmarks [8, 50]. Recently, small molecules targeting RPSA and able to interfere with cell binding to LM, have been identified by in silico techniques. The authors focused on the peptide G of RPSA [27], a sequence of twenty aminoacids that binds both LM and PrP^C^ with high affinity and mediates several cellular events linked to carcinogenesis. The best compound, named NSC47924, resulted cell-permeable and selectively inhibited cell adhesion, migration and invasion of cancer cell lines [27]. Next, the same small inhibitor was used by Sarnataro et al. [28] to control trafficking and interaction between PrP^C^ and RPSA, as well as the activation of “non-canonical” autophagic pathway in neuronal cells [134].

Both PrP^C^ and RPSA show an intrinsically disordered protein region (IDPR) (Fig. 1). It is known that IDPRs are commonly involved in a wide range of biological functions, which complement the ones attributed to ordered and structured protein domains (reviewed in Uversky 2013) [150]. Disordered domains are protein regions that do not assume unique three-dimensional structures and have been found to play different roles in the modulation of binding partners function and in promotion of supra-molecular complexes. Thus, multifunctionality of PrP^C^ and RPSA might be attributed to this characteristic feature which make them involved in a wide range of physiological and pathological roles.

In conclusion, despite evidence related to PrP^C^ and RPSA interaction in cancer are beginning to be unveiled, future investigations may allow the development of therapeutic strategies to target this complex specifically.

## Data Availability

Not applicable.

## Abbreviations

APE1: Apurinic/apyrimidinic Endonuclease 1
APP: Amyloid precursor protein
ATG: Autophagy-related gene
ATM: Ataxia–telangiectasia mutated
Aβ: Amyloid beta
BC: Breast cancer
CAM-DR: Cell adhesion-mediated drug resistance
CAV1: Caveolin 1
CMA: Chaperone-mediated autophagy
CRC: Colorectal cancer
CSCs: Cancer stem-like cells
DDR: DNA damage response
EMT: Epithelial mesenchymal transition
ERK: Extracellular signal regulated kinase
FAK: Focal adhesion kinase
FLNA: Filamin A
GBM: Glioblastoma
GC: Gastric cancer
GPI: Glycosyl-phosphatidylinositol
GPI–PSS: GPI peptide signaling sequence
GSCs: Glioblastoma stem-like cells
HOP: Hsp70–Hsp90-organizing protein
HSPG: Heparan sulphate proteoglycans
hTERT: Human telomerase reverse transcriptase
IDPR: Intrinsically disordered protein region
IGF-1R: Insulin-like growth factor-1 receptor
ILK: Integrin linked kinase
ITGA6: Integrin alpha 6
JNK: Janus kinase
LAC: Lung adenocarcinoma
LC3: Microtubule-associated protein 1A/1B-light chain 3
LM: Laminin-1
LR: Laminin receptor
MAPK: Mitogen activated protein kinase
MMP: Matrix metalloproteinase
mTOR: Mammalian target of Rapamycin
OC: Ovarian cancer
PC: Pancreatic cancer
PDAC: Pancreatic ductal adenocarcinoma
PI3K: Phosphatidyl-inositol-3 kinase
PI3KC3/BECN1: PI3K-class 3/beclin 1
PrP^C^: Cellular prion protein
PrP^Sc^: Scrapie prion protein
RPSA: Ribosomal protein SA
TIMAP: Transforming growth factor-β inhibited membrane associated protein
TME: Tumor microenvironment
